# An Exploratory Study of Strategy Use on Elicited Imitation Tasks

**DOI:** 10.3389/fpsyg.2022.917168

**Published:** 2022-06-17

**Authors:** Yuyun Lei, Xun Yan

**Affiliations:** ^1^Department of East Asian Languages and Cultures, Wake Forest University, Winston-Salem, NC, United States; ^2^Department of Linguistics, University of Illinois Urbana-Champaign, Urbana-Champaign, IL, United States; ^3^Beckman Institute for Advanced Science and Technology, Urbana, IL, United States

**Keywords:** strategy use, elicited imitation, validity, proficiency, Chinese

## Abstract

Elicited imitation (EI) has gained popularity with recent interests in the quest for efficient and flexible measures of second language (L2) proficiency. Despite the surge of interests, questions remain as to what specific linguistic knowledge, skills, and strategies EI measures. To contribute to this line of inquiry, this study explored the nature of strategy use and its effect on EI performance to elucidate the constructs of EI. Twenty-four L2 learners and eight native speakers of Chinese completed an EI test of Chinese and a strategy use questionnaire after the test. Qualitative analyses of the questionnaire responses revealed that participants mainly employed five types of strategies, including approach strategies, cognitive strategies, metacognitive strategies, communication strategies, and test-wiseness strategies. While native speakers reported the least number of strategies, higher-proficiency L2 learners reported more strategies than lower-proficiency L2 learners. We further subjected strategy use, along with participant proficiency level, item length, and item complexity level, to linear mixed-effects regression analyses. The results showed that participant proficiency level, item length, and item complexity level explained the largest test score variance; in contrast, strategy use of different types only accounted for a smaller proportion. The total number of cognitive strategies had a significant, positive effect on EI performance whereas the total number of metacognitive strategies had a significant, negative effect. These findings offer some insights into the nature of speech comprehension and production on EI and provide validity evidence for the use of EI as a language proficiency measure.

## Introduction

With the COVID-19 global pandemic and ongoing quest for efficient, flexible, and accessible instruments to measure second language (L2) proficiency, traditional tasks that elicit constrained responses but can be easily administered online have regained popularity. One of them is the elicited imitation (EI) task. EI, also known as sentence repetition, requires test takers to listen to a series of stimulus sentences and repeat them verbatim (Underhill, 1987). EI has been frequently used as an instrument to measure participants’ proficiency levels in second language acquisition (SLA) research (e.g., Ortega et al., 2002; Gaillard and Tremblay, 2016; Kim et al., 2016). Recently, EI has also appeared in high-stakes, large-scale language proficiency tests, such as the *Versant* tests by Pearson (Bernstein et al., 2010), the *Duolingo English Test* (Ishikawa et al., 2016), and the newly developed *TOEFL Essentials* test (Davis and Norris, 2021). Despite the resurgence of interests in EI, scholars remain dubious about its construct validity (i.e., what it measures) and question the impact of individual differences on EI performance. Previous studies have addressed this concern by examining the role of learners’ memory capacity in EI performance (Okura and Lonsdale, 2012; Kim et al., 2016; Park et al., 2020), finding mostly weak correlations between working memory and EI performance (*r* ranges from 0.25 to 0.31). In these studies, the strongest predictors of learners’ EI performance were shown to be language-related, either their course levels or their performance on another language task. While these studies provide supportive validity evidence for EI as a measure of L2 proficiency, research in this area is still in its infancy to uncover the linguistic constructs measured by EI. According to the *Standards for Educational and Psychological Testing* (AERA, APA, and NCME, 2014, p. 15), “Questioning test takers from various groups making up the intended test-taking population about their performance strategies or responses to particular items can yield evidence that enriches the definition of a construct”. The use of strategies as part of test taker individual characteristics form an important source of validity evidence for understanding the construct of a task. Inspired by this line of inquiry, this study explored individuals’ strategy use and its effect on the language performance of a Chinese EI test. In doing so, we attempt to provide construct validity evidence for EI by elucidating what linguistic knowledge, skills, and strategies EI elicits in speech comprehension and production.

## Literature Review

### Elicited Imitation as a Measure of Second Language Proficiency

EI has been used as a measure of language proficiency for decades in different research domains. It was first used in first language acquisition research (Fraser et al., 1963; Slobin and Welsh, 1973) and later applied to SLA research (Naiman, 1974; Markman et al., 1975). Despite its popularity among SLA researchers, the exact linguistic knowledge and skills measured by EI tasks have not always been clear in the literature. Depending on research design, EI has been claimed to measure L2 listening (Jensen and Vinther, 2003), L2 grammatical knowledge (e.g., Ellis, 2005; Bowles, 2011), L2 lexical development (West, 2012), L2 pronunciation (Trofimovich and Baker, 2007; Yoon, 2010), and L2 oral proficiency (e.g., Ortega et al., 2002). This is largely because EI allows researchers and test developers to construct sentences flexibly to target specific linguistic elements. That said, there is a general consensus among SLA researchers that EI is a measure of general L2 proficiency or L2 oral proficiency. Because EI is dependent on oral production and presented under time pressure, many SLA researchers have further argued that EI is a measure of implicit grammatical knowledge (Ellis, 2005; Erlam, 2006; Bowles, 2011; Spada et al., 2015). Empirical evidence from factor analysis also tends to group EI with other types of tasks that are commonly used to measure implicit knowledge (e.g., timed grammaticality judgment tasks). However, this argument has not been accepted by all scholars. For example, Suzuki and Dekeyser (2015) recently argued that EI does not measure implicit knowledge but instead measures automatized explicit knowledge. Thus, whether EI is a measure of implicit knowledge still requires further investigation. Nevertheless, EI, as a language proficiency measure, has been widely accepted and used in SLA research since the 1970s (Naiman, 1974). Thus far, EI tests have been developed and validated as a measure of L2 proficiency in Spanish (Bowden, 2016), German, Japanese, English (Ortega et al., 2002), French (Tracy-Ventura et al., 2014; Gaillard and Tremblay, 2016), Korean (Kim et al., 2016), Mandarin Chinese (Wu and Ortega, 2013), Russian (Mozgalina, 2015), and Vietnamese (Chaudron et al., 2005).

The theoretical rationale behind EI as an L2 proficiency measure is that in order to repeat a sentence correctly, one has to understand the meaning of the sentence. Since a sentence exceeding the capacity of short-term memory would be difficult to imitate without actual comprehension, a speaker has to decode the sentence using their linguistic knowledge from long-term memory and then reproduce it (Bley-Vroman and Chaudron, 1994). As such, an EI test typically consists of a number of stimulus sentences, ranging in length (measured in syllables, morphemes, or words) and containing a variety of target features (e.g., specific syntactic structures). For L2 learners, when the stimulus sentence goes beyond their current level of the target language, such as containing unfamiliar vocabulary or presenting new grammatical structures, they are likely to repeat only part of the sentence (the part that they can understand) correctly at best or, at worst, fail to repeat the entire sentence. Therefore, EI responses can reveal L2 learners’ strengths and weaknesses in linguistic knowledge and skills, which can facilitate teaching, learning, and other test score uses (Yan et al., 2020). Meta-analysis studies demonstrate that EI, as a general L2 proficiency measure, has a strong ability to discriminate speakers across proficiency levels (Yan et al., 2016; Kostromitina and Plonsky, 2021) and higher reliability compared to other speaking tasks (Henning, 1983).

Although EI as a useful L2 proficiency measure has been accepted by many SLA researchers, there is another concern about its construct validity, that is, whether EI measures language comprehension and production or elicits rote memorization of sounds (Vinther, 2002). Early research observed that when EI stimuli were short enough to be retained as an acoustic representation in short-term memory, it was possible for someone without the knowledge of the target language to “parrot” the stimuli (Fraser et al., 1963; Prutting et al., 1975). This led to the criticism of EI that it measures “perceptual-motor skill” rather than language ability (Fraser et al., 1963, p. 483). To provide evidence for the validity argument of EI as an L2 proficiency measure, a large body of research has focused on establishing the close relationship between L2 learners’ EI performance and their performances on other commonly accepted tests of language proficiency. For instance, Ortega et al. (2002) compared L2 learners’ EI performance with their scores on simulated Oral Proficiency Interview (OPI) and the Test of English as a Foreign Language (TOEFL). Participants’ EI scores showed moderate to strong correlations with the OPI and TOEFL scores (*r* = 0.49 with the TOEFL scores, *r* = 0.61 with the OPI ratings in Japanese, and *r* = 0.88 with the OPI ratings in Spanish). Erlam (2006) found that L2 learners’ EI scores were highly correlated with their scores on subcomponents of the International English Language Testing System (IELTS; *r* = 0.67 with the IELTS speaking score and *r* = 0.72 with the IELTS listening score). Kim et al. (2016) observed similar high correlations between EI scores and the Test of Proficiency in Korean (TOPIK) scores (*r* = 0.62 with the listening score, and *r* = 0.77 with the speaking score). All these results demonstrated that EI, like other established proficiency tests, measures language proficiency of L2 learners.

Another line of research approaches the construct validity of EI by directly investigating the relationship between working memory capacity and EI performance. Okura and Lonsdale (2012) administered an English EI test and a non-word repetition test to sixty-seven students learning English as a Second Language (ESL). They found that EI performances by these participants were significantly correlated with their curricular levels (*r* = 0.79, *p* < 0.001), but not with their scores on the non-word repetition test (*r* = 0.25, *p* = 0.12), which was considered as an index of their working memory capacity. Kim et al. (2016) examined how phonological short-term memory capacity was related to EI performances by sixty-six L2 learners of Korean. A similarly weak correlation was observed between EI scores and phonological short-term memory capacity measured by the digit span test (*r* = 0.30, *p* > 0.01). Park et al. (2020) conducted the same Spanish EI test in Ortega et al. (2002) and a non-word repetition test on seventy-eight L2 learners of Spanish. L2 learners’ EI performances were predicted by their performances on an oral narrative task more than their scores on the non-word repetition task, but they found memory capacity may have a facilitative effect for beginning L2 learners. While these studies demonstrated that EI measures a construct that is different from the one measured by memory tests, the question remains as to what specific linguistic constructs EI measures, that is, what linguistic knowledge, skills, and strategies EI elicits in speech comprehension and production.

### Test-Taking Strategies as Evidence for Construct Validity

In understanding the constructs of a task, one can not only look at “which responses are considered correct” (i.e., product) but also “what process underlies them” (i.e., process; Alderson, 2000, p. 97). As reviewed above, previous research tends to only focus on the products of EI by examining relationships between scores on EI and scores on other tests that measure either similar or distinct constructs. As Cohen (2006) pointed out,

“*what was missing was the aspect of test validation that related to respondents’ behaviours in taking the tests: little was known about what they were actually doing to produce answers to questions and how it corresponds to the abilities one sought to test*” (p. 89).

Analyzing performance strategies or response processes that test takers engage in on test tasks provides another important source of construct validity evidence in validation studies (AERA, APA, and NCME, 2014). More specifically, analyses of test-taking processes or strategies can offer evidence concerning the fit between the elicited processes in the actual performance and the theorized processes tapped by the construct. If the expected processes are elicited, the test is thought to be valid. If alternative processes, irrelevant to the construct, are observed, the validity of the test warrants questions. Following this line of validation research, this study examined test-taking strategies that participants employed on EI tasks to provide construct validity evidence for EI.

Test-taking strategies, as defined by Cohen (2006) are the “consciously selected processes that the respondents used for dealing with both the language issues and the item response demands in the test-taking tasks at hand” (p. 89). There are three types of test-taking strategies—language learner strategies, test management strategies, and test-wiseness strategies. Language learner strategies assist test takers in “operationalizing the targeted language skills” for a task (Cohen, 2013, p. 3). For instance, employing inferencing strategies would be helpful for test takers to respond to some listening comprehension items. Test management strategies allow test takers to respond “meaningfully to the test items and tasks”, such as outlining a plan before speaking. These two types of strategies are expected operations and procedures for task completion and are construct-relevant for test tasks. In contrast, test-wiseness strategies enable test takers to use “knowledge of test formats and other peripheral information to answer test items without going through the expected linguistic and cognitive processes” (Cohen, 2006, p. 90) and thus are considered construct-irrelevant. The degree to which construct-relevant and -irrelevant strategies can be used by test takers determines the validity of a test (Cohen, 2013). Regarding the specific functions of the strategies, test-taking strategies can be mainly classified into approach strategies, cognitive strategies, metacognitive strategies, and communication strategies. According to Swain et al. (2009), approach strategies orient test takers to the task; cognitive strategies involve manipulating the target language to understand and produce language; metacognitive strategies involve organizing, planning, and evaluating language performance; and communication strategies are strategies used for solving a linguistic problem in order to reach a communicative goal.

Test-taking processes or strategies are often obtained by the use of verbal reports in strategy use research. Common verbal reports include think-aloud (e.g., Vandergrift, 1997), stimulated recalls (e.g., Swain et al., 2009), and self-report interviews and questionnaires (e.g., Oxford and Ehrman, 1995). There are some concerns about the veridicality of the verbal report data, that is, whether the data actually reflects participants’ thought processes during task completion. However, researchers have suggested that this threat can be minimized if there is only a short delay between task performance and self-report (Bowles, 2011). In addition, while this type of data may not be exhaustive, it offers a window into the cognitive processes of how test takers arrived at their performance, providing valuable information that cannot be easily addressed by other methods (Gass and Mackey, 2013). More advanced technologies, such as eye-tracking techniques (e.g., Storey, 1997) and event-related brain potentials (e.g., Van Hell and Tokowicz, 2010), have been used in recent strategy use research. Nevertheless, verbal reports remain to be the primary research tool as it is less intrusive and can be effectively conducted.

There has been an increase in recent years in the number of studies investigating test-taking strategies to provide new sources of evidence for construct validity of language tests. For example, Storey (1997) obtained think-aloud data from twenty-five female Chinese students when they were completing an English discourse cloze test. The analyses revealed that participants employed strategies to analyze the rhetorical structure of the text, which supported the argument that cloze tests involve the discourse processing ability, providing validity evidence for the use of cloze tests as a measure of general language ability. Utilizing a strategy inventory, Yang and Plakans (2012) investigated ESL learners’ strategy use and its relationship to test performance on an integrated reading–listening–writing test task. They found that the task requires not only text comprehension and production abilities, but also regulation skills to coordinate reading, listening, and writing materials. These strategies conformed to the strategies proposed in the literature on integrated writing, therefore supporting the valid use of integrated reading–listening–writing tests for assessing academic writing ability. Brunfaut and McCray (2015) used both eye-tracking and stimulated recalls from ESL learners taking the reading proportion of the Aptis test developed by the British Council. Different patterns were observed in the processes used to complete the different reading tasks, including the common lower-level (e.g., lexical access and syntactic parsing) and higher-level (e.g., inferencing and creating paragraph-level representations) reading processes. They also found that participants employed test-wiseness strategies (e.g., the reliance on guessing and background knowledge) to complete the tasks, but the strategies were only used to a limited extent. They concluded that the Aptis reading component adequately taps into the construct of reading skills and thus was a valid reading test.

Some test-taking strategy research involved test takers at different proficiency levels and examined the relationship between strategy use and test performance. Test takers at different proficiency levels have been found to utilize strategy differently (e.g., Green and Oxford, 1995; Oxford and Burry-Stock, 1995; Bruen, 2001). These differences are argued as the result of different proficiency levels and would contribute to differential test performance (e.g., Gordon, 1987; Chamot et al., 1988). A positive relationship has been reported between proficiency level and the use of certain types of strategies, such as metacognitive strategies (e.g., Purpura, 1999; Phakiti, 2003), cognitive strategies (e.g., Oxford and Ehrman, 1995; Park, 1997), and compensation strategies (e.g., Dreyer and Oxford, 1996; Nakatani, 2006). A number of studies also observed that within the same category of strategy, some strategies had positive effects on test performance, while others had negative effects. For example, Song (2005) found that the cognitive strategy of linking with prior knowledge contributed positively to the prediction of the listening and writing scores of the Michigan English Language Assessment Battery (MELAB), while the cognitive strategy of repeating/confirming information showed a negative impact on the MELAB scores. The effect of particular strategy use on test performance may be dependent on task types and contexts.

Compared to the test-taking strategy studies on listening, reading, and writing assessments, the research on strategy use in the assessment of speaking is limited. Yoshida-Morise (1998) identified the communication strategies twelve ESL learners used in their Oral Proficiency Interviews. She found that overall lower-proficiency learners used a higher number of strategies than higher-proficiency learners to compensate for their insufficient L2 knowledge. However, she also observed that higher-proficiency learners used certain communication strategies more than lower-proficiency learners, such as restructuring and repair strategies. In response to the debate over the use of independent and integrated speaking tasks for the assessment of oral proficiency, Swain et al. (2009) and Barkaoui et al. (2013) examined the reported use of strategies based on stimulated recalls from 30 Chinese-speaking engineering students after performing on the independent and integrated speaking tasks of the TOEFL iBT. They found that the integrated tasks involving more language skills elicited a wider variety of reported strategy use than did the independent tasks. Although the total number of reported strategies had no relationship with the total scores on the TOEFL iBT speaking tasks, they argued for the inclusion of both integrated and independent speaking tasks in the assessment of oral proficiency as they elicited different strategy use that tapped into distinct constructs of communicative performance. Similarly, Huang (2013) explored strategies that 40 test takers used when responding to three tasks on the IELTS speaking test, including answering questions, speaking about a topic, and holding a discussion with an examiner. Analysis of both reported strategies elicited from stimulated recalls and observed strategies in production data showed that participants shared some similarities and differences in strategy use across the three tasks. However, there were no significant differences in the reported strategy use between intermediate-level and advanced-level learners. Fernandez (2018) qualitatively analyzed 12 ESL students’ stimulated recalls of the completion of the discussion task on a simulated IELTS speaking test. She found that participants used a great number of metacognitive and cognitive strategies and argued that these strategies are integral to speaking performance. In addition, she observed that some strategies (e.g., analyzing input, planning, and elaborating) contributed positively to the quality of test takers’ responses, while some strategies (e.g., linking to previous knowledge/experience and slowing down) negatively impacted test takers’ performances. Huang (2016) used a self-designed strategy inventory to investigate test-taking strategies used by 215 Taiwanese ESL learners on six speaking tasks for the Test of English for International Communication Speaking Test (TOEIC-S). The tasks included text-reading, picture description, integrated read-to-speak tasks, and independent speaking tasks. Using exploratory factor analysis, he identified three major types of test-taking strategies, that is, communication, cognitive, and affective strategies. Among them, the use of communication and cognitive strategies contributed positively to the TOEIC-S performance. These strategies corresponded to those commonly included in the strategies for real-life oral communication; therefore, they argued that the TOEIC-S test assessed oral communication skills in daily life as intended. While these studies focus on open-ended speaking tasks, little is known about the nature of strategy use on EI tasks and how it affects EI performance. The study thus aims to address the following research questions:

1. What is the nature of strategy use employed by L2 learners of Chinese across proficiency levels on a Chinese EI test?
2. How does strategy use affect language performance on the Chinese EI test by L2 learners of Chinese across proficiency levels?

## Materials and Methods

### Participants

A total of 24 L2 learners of Chinese were recruited for the study. Except that one learner was a native speaker of Japanese, all the participants were native speakers of English (two reported additional native languages, including Spanish and Japanese). They were enrolled in the Chinese language program at a U.S. university: ten in beginning-level classes, seven in intermediate-level classes, and seven in advanced-level classes. Based on the ACTFL (2012) Rating Scale, beginning-level students were equivalent to the levels of Novice Mid to Novice High, intermediate-level students were at Intermediate Low to Intermediate Mid, and advanced-level were at Intermediate High to Advanced Low. There were 13 females and 11 males, with an average age of 20.83. Eight native speakers of Chinese (4 females and 4 males, mean age = 25.5) also participated in this study to provide a baseline measure. They were at the Superior level on the ACTFL Rating Scale.

### Instruments

All the participants completed an EI test of Chinese, a strategy use questionnaire, and a language background questionnaire. The EI test was designed based on a corpus analysis of the widely used Chinese textbooks, the primary source of the language input and use for L2 learners of Chinese in U.S. universities. According to a survey conducted to over 200 universities by the Chinese Language Teachers Association of the U.S. (Li et al., 2014), we compiled a corpus of 36 widely used Chinese textbooks, amounting to a total of 688 unit texts and 703, 995 characters. The corpus was divided into three sub-corpora, corresponding to the three proficiency levels of a typical university-level Chinese program, that is, beginning, intermediate, and advanced. Using corpus analysis techniques, we identified a list of commonly shared vocabulary and grammar (i.e., occurs the most frequently) across the textbooks at each of the three proficiency levels. Based on this list, we designed three sets of EI sentences, each set targeting one of the three proficiency levels. In addition, to reflect the language that L2 learners of Chinese are exposed to, the actual average length of the sentences at each level in the textbook corpus were adopted in the EI item design, which are 8 syllables (beginning and intermediate levels) and 12 syllables (advanced-level). A total of 72 sentences were developed, evenly distributed across the three lexico-grammatical complexity levels at the two lengths bands. Five Chinese language teachers checked the sentences and ensured their naturalness. Following the common practice of administering EI as a proficiency test (Ortega et al., 2002), a 2-s silence was inserted between the end of each sentence and a 0.5-s ringtone prompting the start of the repetition. The EI test went through an iterative process of development and validation and demonstrated good psychometric qualities (Yan et al., 2020).^1^ Sample EI sentences of each level are provided below.

这件衣服非常便宜 (Beginning-level, 8 syllables).

*This piece of clothing is very cheap*.

我每天都坐剬共汽车去学校  (Beginning-level, 12 syllables).

*I take the bus to school every day.*

他比以前进步多了 (Intermediate-level, 8 syllables).

*He made much more progress than before.*

他们的房间里挂着一张地图  (Intermediate-level, 12 syllables).

*There is a map hanging in their room.*

他从事于研究工作 (Advanced-level, 8 syllables).

*He does research for his job.*

他不但不高兴，反而有点生气  (Advanced-level, 12 syllables).

*He is not happy; on the contrary, he is a little angry.*

A questionnaire was used in this study to elicit processes and strategies that participants employed for the completion of the EI test. As this is an exploratory study, open-ended questions were designed to elicit participants’ thoughts on the processes and strategies when they were completing the EI test. Although stimulated recall is frequently used for such purposes (Gass and Mackey, 2013), it could not conveniently be implemented when this study was conducted due to the physical and technical constraints posed by the COVID-19 pandemic. Cohen (2014) suggested that questionnaires can also gain insights into test takers’ strategy use when the questionnaires include information about test takers’ thoughts about their behaviors on test tasks. The main questions probing into test-taking strategies in the questionnaire were:

1. What did you think was/were the key(s) to completing this task?
2. How did you complete this task? Please elaborate your thinking and speaking processes.
3. What do you think makes this task difficult for you? And what did you do to deal with these difficulties?
4. What strategies did you use to help you complete the task? Please provide as many as you can.

The language background questionnaire gathered basic information about participant demographics, language background, and language learning experience. All the tasks were delivered online *via* the Gorilla Experiment Builder (Anwyl-Irvine et al., 2019) to accommodate the need of online testing due to the COVID-19 pandemic.

### Procedure

Each participant received a website link after they signed up for the study. They completed the tasks at their convenience. During the EI test, each participant first listened to a stimulus sentence, waited for two seconds, and then started to repeat the sentence after hearing a ringtone. They were instructed to repeat as much as possible of what they heard. They had 20 s to repeat. After 20 s, a new sentence was automatically played. Each sentence was played only once. Immediately after taking the EI test, participants responded to the strategy use questionnaire to reflect on the processes and strategies they employed when completing the test. In the end, they filled out the language background questionnaire. The total time lasted about 20 to 30 min for each participant.

### Grading and Coding

Two native speakers of Chinese rated all the EI responses using a five-point rating scale (see below, adapted from Ortega et al., 2002). The inter-rater reliability was high, with a value of 0.85 on Cohen’s Kappa. Any disagreements between the two raters were resolved through discussion.

4—Exact repetition or synonymous substitutions;

3—Minor deviation with more than half of the sentence repeated;

2—Half repetition;

1—Inadequate repetition with less than half of the sentence repeated;

0—Silence, unintelligible words, minimal repetition.

Descriptive statistics of the participants’ test scores on the EI test are shown in Table 1. The total test score was 288. Participants performed as expected: native speakers had almost perfect scores; L2 learners from higher curricular levels scored better than learners from lower curricular levels. The level differences were statistically significant [*F*(3, 28) = 28.68, *p* < 0.001, *η*2 = 0.75], indicating that the EI test used in this study was effective at discriminating participants across proficiency levels.

**Table 1:** Descriptive statistics of EI test scores.

Participant level	N	Mean	Median	SD	Lower 95% CI	Upper 95% CI
Beginning	10	116.90	125	46.72	83.49	150.31
Intermediate	7	170.29	161	60.56	114.28	226.30
Advanced	7	229.29	221	27.87	203.52	255.06
Native	8	287.75	288	0.71	287.16	288.34

Participants’ responses to the strategy use questionnaire were qualitatively analyzed to identify strategies participants employed when completing the EI test. We first compiled a list of strategies reported in the questionnaire responses. Then the strategies were tallied for each participant by the two raters, with 1 representing the use of a particular strategy and 0 being the absent use of such a strategy. Adopting taxonomies of strategies in Swain et al. (2009) and Vandergrift (1997), the reported strategies were classified into five main types of strategies: approach, cognitive, metacognitive, communication, and test-wiseness strategies. The exact agreement between the two coders was 95%. Disagreements were also resolved through discussion.

### Statistical Analyses

Descriptive statistics were calculated to examine possible trends of strategy use across proficiency levels. Linear mixed-effects regression analyses were performed to investigate the effects of the use of different strategies on EI performance. The *lm4* package (Bates et al., 2015) in R (version 3.6.1; R Core Team, 2019) was used. The dependent variable was EI score on each item (2,304 data points = 32 participants ^*^ 72 items). Items and participants were treated as random intercepts; participant proficiency level, item length, item complexity level (i.e., targeting beginning, intermediate, or advanced lexico-grammar), and the total number of each type of strategies were treated as fixed effects. The sample size exceeded the minimum recommendation for properly powered mixed-effects models by Brysbaert and Stevens (2018).

## Results

### Strategy Use Across Proficiency Levels

**Figure 1:**
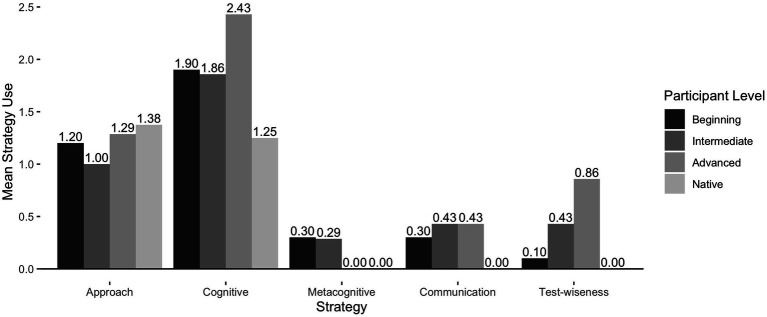
Mean frequencies of strategy use by participant level.

The first research question concerns what strategies were reported using on the EI test by the participants and how these strategies varied across proficiency levels. Table 2 summarizes the frequencies of different types of strategies employed on the EI test. Figure 1 represents the mean frequencies of strategy use across proficiency levels. We found a total of 122 instances of strategy use in participants’ questionnaire responses. The strategies were classified into five main categories of strategies, including approach strategies, cognitive strategies, metacognitive strategies, communication strategies, and test-wiseness strategies. Approach strategies set the goals for participants to complete the EI test. Cognitive strategies help participants better understand and repeat the stimulus sentences. Metacognitive strategies direct participants’ attention to aid the completion of the test. Communication strategies involve conscious plans to deal with linguistic breakdowns. Test-wiseness strategies are strategies of completing the test without understanding the stimulus sentences. Overall, participants reported using cognitive strategies the most frequently (n = 59), followed by approach strategies (n = 39), test-wiseness strategies (n = 10), communication strategies (n = 9), and metacognitive strategies (n = 5) being the least frequent. Among the participants, native speakers reported using the least number of all strategies. As some native speakers mentioned that the process of completing the EI test was very easy for them—listening, memorizing, and repeating, it was not necessary for them to intentionally use strategies to complete the test. Native speakers only used approach strategies to set the goals for completing the test and cognitive strategies to help process and retain the information in the stimulus sentences. In contrast, L2 learners used a variety of strategies to complete the EI test. In addition to approach and cognitive strategies, they used metacognitive, communication, and test-wiseness strategies to deal with the linguistic challenges presented by the EI test. Higher-proficiency L2 learners on average employed a greater number of strategies than did lower-proficiency learners. As shown in Figure 1, there were positive trends of using approach, cognitive, communication, and test-wiseness strategies as the level of proficiency advances among L2 participants, while a negative trend was observed for the metacognitive strategies. In other words, higher-proficiency L2 learners used more approach, cognitive, communication, and test-wiseness strategies than did lower-proficiency learners, but less metacognitive strategies.

**Table 2:** Frequencies of all strategies reported by the participants.

Strategy type	Participant level Raw Frequency (Mean)				Total
	Beginning (*n* = 10)	Intermediate (*n* = 7)	Advanced (*n* = 7)	Native (*n* = 8)	
Approach	12 (1.20)	7 (1.00)	9 (1.29)	11 (1.38)	39
Cognitive	19 (1.90)	13 (1.86)	17 (2.43)	10 (1.25)	59
Metacognitive	3 (0.30)	2 (0.29)	0 (0.00)	0 (0.00)	5
Communication	3 (0.30)	3 (0.43)	3 (0.43)	0 (0.00)	9
Test-wiseness	1 (0.10)	3 (0.43)	6 (0.86)	0 (0.00)	10
Total	38 (3.80)	28 (4.00)	35 (5.00)	21 (2.63)	122

Table 3 presents the frequencies of subcategories of each type of strategy. There were 15 different subcategories of strategies reported by the participants. In the category of approach strategies, the participants employed three different strategies, that is, *getting as many words repeated as possible*, *getting the main message of the sentence out*, and *controlling the accuracy of the language*. Participants at lower-proficiency levels tended to focus more on repeating as many words as possible, while participants at higher-proficiency levels largely focused on the main message of the sentence. One beginning-level participant commented in the questionnaire that she thought the key to completing the test was to comprehend the main message of the sentence, but in actuality, she tried to repeat as many words as possible. This might have been caused by the fact that she could not understand the sentences, so she resorted to the words that she knew. In addition, more higher-proficiency participants tried to control the accuracy of the language they produced. For instance, one advanced-level participant commented below that he refrained from making up words that he did not know and only repeated the words he was familiar with.

**Table 3:** Frequencies of individual strategies reported by the participants.

Individual strategy	Participant LevelRaw Frequency (Mean)				Total
	Beginning (*n* = 10)	Intermediate (*n* = 7)	Advanced (*n* = 7)	Native (*n* = 8)
**Approach strategies**					
1. Getting as many words repeated as possible	7 (0.70)	3 (0.43)	2 (0.29)	3 (0.38)	15
2. Getting the main message of the sentence out	4 (0.40)	3 (0.43)	5 (0.71)	6 (0.75)	18
3. Controlling the accuracy of the language	1 (0.10)	1 (0.14)	2 (0.29)	2 (0.25)	6
**Cognitive strategies**					
4. Comprehending the meaning of the sentence	6 (0.60)	3 (0.43)	6 (0.86)	5 (0.63)	20
5. Recognizing familiar words, phrases, or structures	5 (0.50)	4 (0.57)	4 (0.57)	0 (0.00)	13
6. Listening for key words, phrases, or structures	2 (0.20)	3 (0.43)	1 (0.14)	1 (0.13)	7
7. Translating	2 (0.20)	1 (0.14)	3 (0.43)	0 (0.00)	6
8. Rehearsing the sentence before repetition	1 (0.10)	2 (0.29)	2 (0.29)	1 (0.13)	6
9. Connecting to daily scenarios	0 (0.00)	0 (0.00)	1 (0.14)	3 (0.38)	4
10. Chunking the sentence into smaller parts	3 (0.30)	0 (0.00)	0 (0.00)	0 (0.00)	3
**Metacognitive strategies**					
11. Prioritizing certain parts of the sentence	3 (0.30)	2 (0.29)	0 (0.00)	0 (0.00)	5
**Communication strategies**					
12. Paraphrasing	1 (0.10)	1 (0.14)	2 (0.29)	0 (0.00)	4
13. Using lexical fillers	1 (0.10)	1 (0.14)	1 (0.14)	0 (0.00)	3
14. Guessing	1 (0.10)	1 (0.14)	0 (0.00)	0 (0.00)	2
**Test-wiseness strategies**					
15. Imitating the sounds	1 (0.10)	3 (0.43)	6 (0.86)	0 (0.00)	10

*For more challenging sentences, I tried to pick up enough familiar words from what the speaker was saying so that I could put together a complete sentence. I tried not to make up any words or assume that I knew what the speaker was saying - instead, for the majority of my responses, I tried to limit what I repeated to the words that I understood* (s19, advanced-level).

As EI involves auditory processing of the stimulus sentences, participants reported using a great number of cognitive strategies. Among the cognitive strategies, *comprehending the meaning of the sentence* was used the most frequently, followed by *recognizing familiar words, phrases, or structures*. Similar to the orientation toward words versus the main message as suggested by the use of approach strategies, lower-proficiency learners reported more strategies on *recognizing familiar words, phrases, or structures*, while higher-proficiency learners focused more on *comprehending the meaning of the sentence*. None of the native speakers employed the strategy of *recognizing familiar words, phrases, or structures*. In addition, more lower-proficiency learners reported using the strategy of *listening for key words, phrases, or structures* to process and understand the information in the sentences. Below are four examples, one from each proficiency level, where participants indicated that they paid attention to key words, phrases, or structures in the sentences while listening. As shown in the examples, higher-proficiency participants were able to focus on larger linguistic units, such as sentence structures, as opposed to lower-proficiency participants who tended to listen for key words or phrases.

*I tried to…listen for phrases I knew, such as the order of subject, time, to whom, and then verb* (s04, beginning-level).

*Listen to the person’s pauses and when they emphasize certain words and phrases* (s06, intermediate-level).

*I focused on thinking about the sentence grammatical structures* (s19, advanced-level).

*I memorized the sentence structure and main verbs* (c07, native).

To retain the meaning of the EI sentences, the *translating* strategy was also utilized by the L2 learners. They translated what they heard into English and then translated it back into Chinese when repeating. Advanced-level learners utilized this strategy more than did the other two levels of learners, as advanced-level learners were more likely to understand the meaning of the sentences. Some participants also used the strategy *rehearsing the sentence* before the start of the repetition to help them remember the information. Since rehearsing without comprehension would be difficult, this strategy was used more frequently by higher-proficiency participants. As they were able to comprehend the meaning of the sentences, higher-proficiency participants also used the strategy *connecting to daily scenarios* to help retain the information (see response examples below). For some beginning-level learners, the sentences in the test might have been too long for them to process. Therefore, they reported using the strategy *chunking the sentence into smaller parts* to help themselves understand and memorize the parts they could understand.

*I tried to imagine someone saying that to me in real life and tried to listen to the whole sentence to understand it rather than memorizing it* (s24, advanced-level).

*Sometimes I constructed some scenarios based on what I heard. For example, 这家饭馆的菜不如那家的好 (The food in this restaurant is not as good as the other restaurant). It feels like a sentence you would say when you go out to eat with your friends* (c03, native).

As regards metacognitive strategies, we found that participants employed one strategy *prioritizing certain parts of the sentence* to complete the test. Only beginning- and intermediate-level L2 participants reported using such a strategy. Since the beginning or the last few words in the sentences are easy to be held in short-term memory, some beginning- and intermediate-level participants prioritized either the beginning or the end part of the sentences in order to complete the test. Two response examples are provided below.

*Chunk the info as it was coming in, use beginning only* (s13, beginning-level).

*If the sentence was long and in multiple parts, especially if I did not know what it meant, I tried to retain the last part of it, because since it was right before I had to repeat it, I found I could remember it better than the first part* (s01, intermediate-level).

In the category of communication strategies, we found that L2 participants employed three communication strategies to compensate for areas where they experienced linguistic breakdowns, including *using lexical fillers*, *paraphasing*, and *guessing*. When L2 participants encountered places where they could not understand or remember the words, they used lexical fillers, such as “something” or “什么” (“something” in Chinese), to fill in the gaps (see examples 1 and 2 below). Moreover, some participants could understand the syntactic structures of the sentence and fill the gaps with the words that correspond to the missing part of speech, as shown in example 3.

1. 今年冬天很冷，可是没有下雪 (stimulus sentence)

*Winter in this year was very cold, but it did not snow.*

今年something很冷，可是没有下雪 (s06, intermediate-level).

*Something in this year was very cold, but it did not snow.*

2. 图书馆在剬园旁边 (stimulus sentence)

*The library is next to the park.*

图书馆在什么旁边 (s11, advanced-level).

*The library is next to something.*

3. 晚上我想跟朋友一起去跑步 *(stimulus sentence)*

*Tonight I want to run together with my friend.*

什么什么时候我和我朋友去跑步 (s21, beginning-level).

*A time I run with my friend.*

A few L2 participants said that sometimes they would paraphrase in their responses when they understood the meaning of the sentence but could not remember the exact words in the sentence. As shown in the example 4, the L2 participant understood the general gist of the sentence, but probably missed the degree modifier for the adjective. Therefore, he replaced the word “非常” (very) with “真” (really) in his response. Two lower-level L2 participants reported that they guessed either the structure of the sentence based on the phrasing or a few words after the part they recognized.

4. 这件衣服非常便宜 (stimulus sentence)

*This piece of clothing is very cheap.*

这件衣服真便宜 (s23, advance-level).

*This piece of clothing is really cheap.*

We found that L2 participants employed one type of test-wiseness strategy *imitating the sounds* on the EI test. Many L2 learners at higher-proficiency levels reported that they employed two approaches to complete the EI test. They first tried to comprehend the meaning of the sentence. If they could understand it, they would repeat what they heard. If they could not understand it, they would imitate the sounds of the words. A response example is provided below.

*If I understood what was said, it was not hard for me to just recreate it in my head and repeat it. However, if there were phrases or words used that I had never heard before, I sometimes had to refer solely to my auditory memory to try and repeat what was said* (s11, advanced-level).

Unlike guessing that involves certain levels of L2 processing based on known information, imitation of sounds relies solely on participants’ auditory memory. This strategy is considered to be construct-irrelevant. There were more L2 learners at higher-proficiency levels reported conscious use of this strategy.

Overall, there was a relative increase in frequencies across proficiency levels for the following strategies: approach strategies of *getting the main message of the sentence out* and *controlling the accuracy of the language*, cognitive strategies of *comprehending the meaning of the sentence*, *recognizing familiar words, phrases, or structures*, *translating*, *rehearsing the sentence before repetition*, and *connecting to daily scenarios*, communication strategies of *paraphrasing* and *using lexical fillers,* and the test-wiseness strategy *imitating the sounds*.

### Strategy Use and Test Performance

To address the second research question about the effects of strategy use on the EI test performance, we conducted linear mixed-effects regression analyses to examine whether the total number of different types of strategies contributed to the EI scores. The dependent variable was EI score on each individual item, and item and participant served as random effects. Table 4 presents the statistics of the linear mixed-effects models. First, an empty model was performed to evaluate the appropriateness of treating item and participant as random effects (see Model I statistics in Table 4). The empty model included only the random effects. The intercepts of the random effects varied considerably between the items (σ^2^ = 0.29, SD = 0.053) and between the participants (σ^2^ = 1.13, SD = 1.06), suggesting the need to treat these two variables as random effects. Next, participant proficiency level, item length, and item complexity level were added to the empty model to examine the main effects of participants’ language ability and task features on the EI scores (Model II). The results of the comparison between the two models showed an improvement in Model II (Δ −2LL = 117.79, Δ*df* = 6, *p* < 0.001). Lastly, total numbers of the five different types of strategies were added to the model to evaluate the effects of strategy use on the EI performance (Model III). Model III yielded a better fit (Δ −2LL = 13.84, Δ*df* = 5, *p* = 0.02), thus it was used as the final model. The final model explained 69.2% of the EI score variance. Participant proficiency level, item length, and item complexity level accounted for 51.5% of the variance, whereas total numbers of different types of strategies accounted for 4.8% of the variance. Participants at higher-proficiency levels performed better than lower-proficiency participants: native speakers had on average higher scores (*β* = 2.37, *p* < 0.001) than advanced-level learners, advanced-level learners received higher scores (*β* = 1.56, *p* < 0.001) than intermediate-level learners, and intermediate-level learners scored higher (*β* = 0.74., *p* < 0.001) than beginning-level learners. Overall the participants had lower performance when repeating EI sentences with 12 syllables (*β* = − 0.53, *p* < 0.001) and targeting intermediate-level (*β* = − 0.39, *p* < 0.001) and advanced-level lexico-grammar (*β* = − 0.86, *p* < 0.001). Among the different types of strategies, the total number of cognitive strategies and metacognitive strategies had significant effects on the EI performance, with cognitive strategies having a positive effect (*β* = 0.27, *p* = 0.002) and metacognitive strategies having a negative effect (*β* = − 0.62, *p* = 0.008). In other words, higher-proficiency learners employed significantly more cognitive strategies, whereas lower-proficiency learners employed significantly more metacognitive strategies. Although other strategies did not yield statistical significance, participants who used more communication strategies (*β* = 0.26, *p* = 0.109) and approach strategies (*β* = 0.18, *p* = 0.228) were likely to have better performance on the EI test, but not if using test-wiseness strategies (*β* = − 0.12, *p* = 0.619).

**Table 4:** Statistics for the linear mixed-effects models: Models I, II, and III.

		Model I: Empty			Model II: Main			Model III: Main + Strategy use		
		*β*	SE	Sig.	*β*	SE	Sig.	*β*	SE	Sig.
Intercept		2.72	0.20	^***^	2.31	0.18	^***^	1.69	0.30	^***^
*Fixed effects*										
Participant level	Intermediate		0.74	0.26	^***^	0.78	0.23	^***^
	Advanced		1.56	0.26	^***^	1.27	0.28	^***^
	Native		2.37	0.25	^***^	2.40	0.23	^***^
Item length: 12 syllables			−0.53	0.07	^***^	−0.53	0.07	^***^
Item level	Intermediate		−0.39	0.10	^***^	−0.39	0.10	^***^
	Advanced		−0.86	0.10	^***^	−0.86	0.10	^***^
Approach strategies				0.18	0.15	0.228
Cognitive strategies				0.27	0.09	0.002^**^
Metacognitive strategies				−0.62	0.24	0.008^**^
Communication strategies				0.26	0.16	0.109
Test-wiseness strategies				−0.12	0.23	0.619
*Random effects*		Variance	SD		Variance	SD		Variance	SD	
Participant		1.13	1.06		0.27	0.52		0.17	0.41	
Item		0.29	0.53		0.09	0.30		0.09	0.30	
−2^*^loglikehood		5821.6			5703.7			5689.9		

*Participants *n* = 32, items *n* = 72*.

** *p* *≤ 0.01 and*

*** *p ≤ 0.001*.

## Discussion

This study investigated the nature of strategy use and its effect on the language performance of a Chinese EI test to provide construct validity evidence for EI tasks. Descriptive statistics revealed that participants used 15 different individual strategies on the EI test, representing five main strategy categories, that is, approach strategies, cognitive strategies, metacognitive strategies, communication strategies, and test-wiseness strategies. The cognitive strategy category was used the most frequently by the participants, followed by approach strategies, test-wiseness strategies, communication strategies, and metacognitive strategies. Most participants set the goals of either *getting as many words repeated as possible* or *getting the main message of the sentence out* to complete the EI test. When they were completing the test, they used cognitive strategies (e.g., *understanding the meaning of the sentence*, *recognizing familiar words, phrases, or structures*, or *translating*) to comprehend and retain the information in the sentences. L2 participants also used the metacognitive strategy *prioritizing certain parts of the sentences* to facilitate the completion of the test and utilized communication strategies (e.g., *using lexical fillers* and *paraphrasing*) to deal with any breakdowns. Since the EI test presented no difficulties for native speakers (as shown by their perfect scores on the test), there was no need for them to employ the reported metacognitive and communication strategies. Native speakers only used approach strategies to set the goals for the test and cognitive strategies to help them comprehend and memorize the sentences. In contrast, L2 participants employed a greater and wider range of strategies to compensate for their insufficient L2 knowledge when completing the EI test. Similar to previous research that higher-proficiency L2 learners were more aware of the strategies they used and why they used them (Chamot et al., 1988; Green and Oxford, 1995), higher-proficiency L2 participants in this study also recalled more conscious use of strategies and how they employed the strategies to complete the test.

Among all the reported strategies, many of these strategies could be found in the test-taking strategies employed for completing common listening and speaking tasks. For example, L2 learners used strategies of *translating*, *paraphrasing*, *using lexical fillers*, and *guessing* when responding to independent and integrated speaking tasks (e.g., Swain et al., 2009). They used strategies of *listening for key words*, *connecting to daily scenarios*, *translating* when completing listening comprehension tasks (e.g., Vandergrift, 1997). Other strategies, such as *understanding the meaning of the sentence* and *recognizing familiar words, phrases, or structures*, involve the operation of listening and speaking skills as well as the entailed language processing (e.g., the comprehension of certain vocabulary or grammar); thus, they are expected strategies relevant to the EI construct. In addition, lower-proficiency participants tended to focus on individual words, whereas higher-proficiency participants were able to deal with sentence-level processing. This aligns with previous observations of the different ways of processing by L2 learners at various proficiency levels on listening and speaking tasks (Buck, 2001; Field, 2011). These findings provide evidence to suggest that EI taps into the processes of comprehending and reproducing speech in ways similar to other listening and speech tasks.

That said, due to the repetitive nature of EI, we also observed a test-wiseness strategy unique to EI, especially among higher-proficiency learners. That is, L2 participants tried to imitate the sounds of the words without comprehension after they failed to understand the sentences. Since this strategy does not require linguistic skills and knowledge of the target language, it can be considered construct-irrelevant. This observation seemed to support some researchers’ concerns that EI elicit mere rote memorization of individual sounds. However, a negative trend of using the test-wiseness strategy on the EI performance was found in the linear mixed-effects analyses. This indicates that the utilization of the test-wiseness strategy generally does not help participants receive higher scores on the EI test. Based on our observation in the data, many of the imitated sounds were unintelligible. These suggest that test users need not be overly concerned about the effect of this test-wiseness strategy on EI score interpretation.

This study also addressed whether strategy use had effects on EI test performance. Linear mixed-effects models indicate that the total number of cognitive strategies used had a significant, positive effect on the EI scores, while the total number of metacognitive strategies had a significant, negative effect on the EI scores. In other words, more strategies pertaining to the meaningful processing and understanding of the sentence would help participants receive higher scores on the EI test. In contrast, more strategies prone to the reliance on the short-term memory would lead to lower scores on the EI test. As reported by some of the beginning- and intermediate-level participants, they prioritized either the beginning or the end part of the sentences when completing the test. Since the beginning or the last few words in the sentences are easy to be held in short-term memory, it is possible that the participants repeated the words without actually understanding the meaning of those words. However, the negative effect of the metacognitive strategies on EI performance provides evidence in support of the use of EI for assessing general language proficiency, as only strategies require the use of linguistic knowledge and skills in the target language would lead to better performance on the test.

Moreover, we found that the greatest proportion of EI score variance was explained by participants’ proficiency levels and item difficulty levels, while strategy use only contributed to a small proportion of EI score variance. Bachman (1990) pointed out that the factors affecting performance on language tests are language ability, individual characteristics of test takers, characteristics of the test method or test tasks, and error of measurement. Among the three types of systematic sources of variability, language ability was the central factor accounting for the variation of test scores. This is consistent with the main effect of proficiency level on EI performance found in the study. This lends support to EI as an indicator of participants’ language proficiency. In addition, previous studies suggested that sentence complexity and sentence lengths are two major factors contributing to the difficulty levels of EI items (e.g., Ortega et al., 2002; Graham et al., 2010; Yan et al., 2016). L2 participants had lower performances when the sentence length increased and when the sentence contained advanced vocabulary and grammar. This further supports that it requires a sufficient level of language proficiency to be able to perform well on the EI test. Moreover, as strategy use is only one part of the characteristics of test takers, it makes sense that strategy use only accounted for a small proportion of EI score variance.

Taken together, the findings suggest that EI scores are valid indicators of participants’ language proficiency. The use of strategies pertaining to the processing and comprehending of the sentences are integral to the successful completion of the EI test, while strategies rely on rote memorization are marginal and detrimental to the test performance.

## Conclusion

This study examined the nature of strategy use and its effect on the language performance of a Chinese EI test. The results revealed that both L2 and native speakers of Chinese utilized different types of strategies to comprehend and reproduce the stimulus sentences in order to complete the EI test. There are some overlaps between the reported strategies and the common strategies test takers would use for listening and speaking tasks. In addition, cognitive strategies that help the processing of the stimulus sentences contributed positively to the performances on the EI test, whereas metacognitive strategies that allow to focus on only a small chunk of the stimulus sentences contributed negatively to the EI performances. Although some L2 participants imitated the sounds without comprehension, this test-wiseness strategy only had a small effect and generally did not contribute to higher scores on the EI test. These findings provide validity evidence that EI taps into the processing and production of the target language rather than rote memorization of individual sounds.

As an exploratory study, this study utilized a questionnaire to elicit strategy use on EI tasks. Although questionnaires are useful in obtaining a general understanding of test-taking processes and strategies, they could only elicit a partial list of all strategies. It is possible that some strategies participants employed on the EI test were not reported in the questionnaires. To further understand test-taking processes and strategies on EI tasks, other forms of verbal reports, such as stimulated recall, can be conducted to reveal more details on the strategy use for EI tasks. The study observed certain overlaps of strategies between EI and integrated listen-to-speak tasks, both of which requires listening and speaking skills. Future research on the comparison of strategies employed on EI versus integrated listen-to-speak tasks may be helpful to elucidate the linguistic constructs measured by EI. Moreover, this study only examined strategy use by a small number of participants. Although small sample size is common for studies on less commonly taught languages in the US, such as Chinese, a more generalizable conclusion can benefit from a larger number of participants.

The limitations notwithstanding, this study offered a process-oriented perspective to understand the constructs of EI tasks. The findings in this study can provide supporting evidence for the construct validity of EI. That is, EI, as a valid language proficiency measure, assesses speech comprehension and production of the target language.

## Data Availability Statement

The elicited imitation test of Chinese used in this study can be found in online repositories. The names of the repository/repositories and accession number(s) can be found at: IRIS (https://www.iris-database.org/iris/app/home/detail?id=york:938753).

## Ethics Statement

The studies involving human participants were reviewed and approved by the Institutional Review Board Office at the University of Illinois at Urbana-Champaign. The patients/participants provided their written informed consent to participate in this study.

## Author Contributions

YL designed the study, performed the experiment, analyzed the data, and wrote and edited the manuscript. XY supervised the research, reviewed the results, provided feedback, and revised the manuscript. All authors contributed to the article and approved the submitted version.

## Funding

This article was supported by Wake Forest University’s Open Access Publishing Fund.

## Conflict of Interest

The authors declare that the research was conducted in the absence of any commercial or financial relationships that could be construed as a potential conflict of interest.

## Publisher’s Note

All claims expressed in this article are solely those of the authors and do not necessarily represent those of their affiliated organizations, or those of the publisher, the editors and the reviewers. Any product that may be evaluated in this article, or claim that may be made by its manufacturer, is not guaranteed or endorsed by the publisher.

## Footnotes

^1^The full test is available on IRIS (https://www.iris-database.org/iris/app/home/detail?id=york:938753), including test items, test instructions, item recordings, and the grading rubric.
